# Integration of LC-HRMS and ^1^H NMR metabolomics data fusion approaches for classification of Amarone wine based on withering time and yeast strain

**DOI:** 10.1016/j.fochx.2024.101607

**Published:** 2024-07-02

**Authors:** Pier Paolo Becchi, Veronica Lolli, Leilei Zhang, Francesco Pavanello, Augusta Caligiani, Luigi Lucini

**Affiliations:** a Department for Sustainable Food Process, Università Cattolica del Sacro Cuore, Via Emilia Parmense 84, 29122 Piacenza, Italy; b Department of Food and Drug, University of Parma, Parco Area delle Scienze 27/A, 43124 Parma, Italy; c 4consulting, via Alberto Dominutti 20, 37135, Verona, Italy; d CRAST research centre, Università Cattolica del Sacro Cuore, Via Emilia Parmense 84, 29122 Piacenza, Italy

**Keywords:** Wine profiling, Multi-omics, Supervised modelling, Data fusion, ^1^H NMR, LC-HRMS

## Abstract

Two untargeted metabolomics approaches (LC-HRMS and ^1^H NMR) were combined to classify Amarone wines based on grape withering time and yeast strain. The study employed a multi-omics data integration approach, combining unsupervised data exploration (MCIA) and supervised statistical analysis (sPLS-DA).

The results revealed that the multi-omics pseudo-eigenvalue space highlighted a limited correlation between the datasets (RV-score = 16.4%), suggesting the complementarity of the assays. Furthermore, the sPLS-DA models correctly classified wine samples according to both withering time and yeast strains, providing a much broader characterization of wine metabolome with respect to what was obtained from the individual techniques. Significant variations were notably observed in the accumulation of amino acids, monosaccharides, and polyphenolic compounds throughout the withering process, with a lower error rate in sample classification (7.52%).

In conclusion, this strategy demonstrated a high capability to integrate large omics datasets and identify key metabolites able to discriminate wine samples based on their characteristics.

## Highlights

• LC-HRMS and ^1^H NMR profiling were applied to 80 Amarone wine samples.
• Both LC-HRMS and ^1^H NMR datasets provided classifications based on the withering time and yeast.
• Unsupervised and supervised multi-omics data fusion approaches were performed on datasets.
• Data fusion approach improved the predictive accuracy of the overall fingerprint of Amarone.

## 1 Introduction

Over the last few years, wine consumers have been increasingly aware of the quality of wine, whose perception is mainly associated with the region, the denomination of origin as well as health and sustainability expectations (Castellini & Samoggia, 2018; Chen et al., 2024).

Among high quality Italian wines, “Amarone della Valpolicella” is a "Controlled and Guaranteed Designation of Origin" (DOCG) red wine produced in the province of Verona (Veneto region of Northeast Italy). Its production is strictly regulated by disciplinary rules which define the types and percentages of indigenous grape varieties, the permitted municipalities for cultivation, the maximum yield per hectare, and the winemaking practices (De Rosso, Mayr, Girardi, Dalla Vedova, & Flamini, 2018). These parameters collectively contribute to the complex combination of thousands of compounds that characterize this wine (Dall'Asta, Cirlini, Morini, & Galaverna, 2011; Samaniego Solis, Luzzini, Slaghenaufi, & Ugliano, 2024).

In recent years, in addition to the analytical method targeting specific molecules, a fingerprinting approach has emerged for food characterization, including wine classification (Mascellani et al., 2021; Prandi et al., 2022). In this progression, untargeted metabolomics via Liquid Chromatography coupled with High-Resolution Mass Spectrometry (LC-HRMS) or Nuclear Magnetic Resonance (NMR) can provide complex fingerprints and accurate quantification of several compounds in a single analysis step, without requiring a specific sample preparation procedure (Gougeon, da Costa, Richard, & Guyon, 2019). Furthermore, these analytical methods can be combined with chemometrics to establish robust databases serving as references for contents, varietals, and regions of production.

The LC-HRMS analysis emerges as a powerful technique for studying grape metabolomics. Through screening grape extracts using this method, researchers have identified new polyphenols (e.g., stilbenes, flavonols, anthocyanins) and aroma precursors (such as monoterpene glycosides), which contribute to the construction of databases containing over a thousand compounds found in grapes and wine (Flamini & De Rosso, 2015; Ueda et al., 2023). At the same time, ^1^H NMR spectroscopy has been widely applied to characterize the origin of wine, its variety and winemaking practices (Gougeon et al., 2019). Recently, a 400 MHz NMR spectrometer-based automated quality control analysis developed by Brucker (https://www.bruker.com/it/resources/library/application-notes-mr/bruker-nmr-foodscreener-aboratory-achieves-iso-iec17025-accreditation.html) has achieved the ISO/IEC 17025 accreditation for wine profiling. This method is now officially recognized by the International Organization of Vine and Wine (www.oiv.int) marking significant progress toward the official standardization of analytical methods based on NMR metabolomics fingerprinting.

By integrating information from various analytical techniques, such as spectroscopy and chromatography, a more comprehensive understanding of food quality attributes can be achieved, improving discriminant performance for sample classification than a single technique (Li, Xiong, & Min, 2019.). Indeed, multi-data approaches, like data fusion, have shown significant promise for enhancing food quality control (Rivera-Pérez, Romero-González, & Garrido Frenich, 2023). However, studies reporting on data fusion in wine profiling are currently limited.

In the present research, a supervised multi-omics data fusion approach for processing outputs obtained from LC-HRMS and ^1^H NMR datasets was performed to provide further insight into wine profiling and unravel the effect of key technological parameters affecting the quality of the wine. With this regard, Amarone wine was used as a case study because of its recognized market value and its complex and articulate winemaking process (Paronetto & Dellaglio, 2011). First, this work presents an investigation by LC-HRMS and ^1^H NMR metabolic profiling combined with multivariate models of single datasets for Amarone classification, based on two main factors for winemaking in determining unique metabolic profile (Dellaglio, Zapparoli, Malacrinò, Suzzi, & Torriani, 2003; Samaniego Solis et al., 2024): grape withering time and yeast strain. Then, a supervised data fusion from the previously reported LC–HRMS and ^1^H NMR datasets was chosen to demonstrate a higher ability to define authentic Amarone wine. This work's rationale is that complementary/synergic effects might arise from combining different multi-source information on wine profiling, as previously postulated for other analytical platforms (Ríos-Reina, Azcarate, Camiña, & Goicoechea, 2020), including other matrices (Rivera-Pérez et al., 2023).

## 2 Materials and methods

### 2.1 Reagents, standards, and materials

LC-MS grade water and acetonitrile were supplied by Sigma-Aldrich (Madison, CA, USA). Deuterium oxide (D_2_O, CAS. N. 7789-20-0, 99.86% D) was obtained from VWR International BVBA (Geldenaakseban, Leuven, Belgium), and 3-(Trimethylsilyl)-2,2,3,3-tetradeutero-propionic acid sodium salt (TSP, CAS No. 24493–21-8, 99% D), oxalic acid (CAS No. 144–62-7, 98%) and sodium oxalate (CAS No. 62–76-0, ≥99.5%) were obtained from Sigma-Aldrich (Milano, Italy). An Eppendorf 5810R centrifuge (Hamburg, Germany) was performed for sample treatment.

### 2.2 Experimental design, winemaking, and sampling

A total of 80 distinct Amarone wine samples were produced by blending the allowed grape varieties (such as cv. Corvina, cv. Corvinone, cv. Rondinella, cv. Merlot, cv. Molinara) as specified by the Product Specifications of Amarone wine production (http://catalogoviti.politicheagricole.it/scheda_denom.php?t=dsc&q=1004,) from ten vineyards located in Valpolicella terroirs (Veneto, Italy) harvested in September 2021. Specifically, each vineyard provided wine samples from grapes that had undergone to four time points in the withering process: (i) before withering; (ii) after 1/3 of withering; (iii) after 2/3 of withering; (iv) end of withering. The first grape sampling (t1) occurred a few hours after the harvesting once the grapes were transferred from the vineyard to the warehouse. The other time point sampling occurred on the 24th, 52nd, and 82nd day following the beginning of withering (herein, time points t2, t3, and t4, respectively). At each time point, must samples were collected as soon as the grapes were mashed using sterile tools to prevent alteration. The duration and the technical parameters of grape withering were defined according to the regional rules for Amarone production (Paronetto & Dellaglio, 2011). The winemaking process of musts was carried out thanks to Italiana Biotecnologie (Trecate, Novara, Italy) in sterile bottles of 0.350 L. Specifically, two different yeast strains were selected for wine fermentation process, namely Inverno 1936 (*Saccharomyces cerevisiae)* and Vulcano (*Saccharomyces cerevisiae* var. *bayanus*), both produced by EVER industry (Treviso, Italy), tested on both withering times under investigation (with a total of 20 samples for each time point). These cryotolerant yeasts are recognized for their reliability during the fermentation process, their high alcohol tolerances, and their proven physiological characteristics, emphasizing the varietal and terroir character of Amarone wine aroma (Dellaglio et al., 2003). However, comprehensive information about the yeast strains employed in this study is available on the manufacturer's website (https://www.ever.it). At the end of fermentation, wine samples were collected in sterile tubes and then stored at −20 °C until analysis by the two techniques described below. Notably, our analyses were performed on Amarone wine without considering the two-year aging period specified in the product guidelines. Our primary aim was to investigate how varying degrees of grape withering and using different yeast strains could directly impact the overall profile of Amarone wine samples. The detailed list of Amarone wine samples is reported in Table S1. Overall, due to the robustness of the study and of the wine sampling process, individual biological samples served as biological replicates for each investigated factor, allowing us to better represent the biological variability within Amarone wine matrix.

### 2.3 Instrumental Analysis

#### 2.3.1 LC-HRMS

In the extraction process for the LC-HRMS analysis, wine samples were thawed at room temperature and extracted in triplicate. Briefly, an aliquot of 850 μL of each sample was added to 850 μL of acetonitrile acidified with 1% (*v*/v) formic acid, then processed with ultrasounds for 10 min and centrifugated at 12000 x *g* at 4 °C for 15 min. Finally, the supernatants were filtered using 0.22 μm cellulose syringe filters and transferred into amber vials for instrumental analysis. Moreover, a quality control (QC) sample was prepared by combining 10 μL from each of the 80 Amarone wine samples into the same UHPLC vial. During the chromatographic run, the QC sample was injected throughout the sequence, specifically at the beginning, every ten analyzed samples and at the end of the batch.

The untargeted profiling was performed through ultra-high-performance liquid chromatography (UHPLC) coupled to a quadrupole-orbitrap mass spectrometer through a heated electrospray ionization (HESI)-II probe (Thermo Scientific, USA). The metabolomic platform consisted of a Vanquish ultra-high-performance liquid chromatography pump coupled to Q-Exactive™ mass spectrometer (Thermo Scientific, Waltham, MA, USA). The instrumental conditions were previously defined (Rocchetti, Michelini, Pizzamiglio, Masoero, & Lucini, 2021). Briefly, the chromatographic separation of wine compounds was performed in reverse phase using a BEH C18 (2.1 × 100 mm, 1.7 μm) analytical column maintained at 35 °C. The mobile phase consisted of ultrapure water (A) and acetonitrile (B), with formic acid (0.1% *v*/v) as a phase modifier. The separation was achieved with an elution gradient mode starting from 6% to 94% B in 32 min with a flow rate of 200 μL/min. For each prepared wine extract, the injection volume was 6 μL. The analysis was conducted in positive polarity (ESI+) using full scan MS in the *m*/*z* range of 100–1200, using a nominal mass resolution of 70,000 FWHM at m/z 200. Furthermore, data-dependent MS/MS approach was only performed on QC spectra during the entire analysis, with precursor fragmentation in positive polarity on the most abundant ions (Top N ions =3) with a reduced full scan mass resolution of 17,500 at m/z 200. In this regard, to induce molecular fragmentation, stepped collisional energies were set at 10, 20, and 40 eV. Regarding the positive electrospray ionization, the overall settings were previously optimized (Rocchetti et al., 2021). The raw data were subjected to pre-processing and data filtering procedures utilizing MS-DIAL software (version 4.80) (Tsugawa et al., 2015). Detailed information regarding the parameter settings for untargeted metabolomics analysis using MS-DIAL can be found in a previously published work. Identification of metabolites involved accurate mass tolerance (5 ppm), isotopic pattern analysis, and spectral matching, with a confidence threshold set at >60% for annotation. The comprehensive FooDB database (foodb.ca) was used for metabolite annotation in the context of wine analysis. Furthermore, to enhance the confidence of annotations, the spectral information of QC samples was employed for additional identification and confirmation steps, using publicly available MS/MS experimental spectra in the MS-DIAL software (Mass Bank of North America).

#### 2.3.2 ^1^H NMR

A simple pre-treatment was carried out on wine samples for ^1^H NMR analysis according to a previous protocol, slightly adapted (Musio et al., 2020): 350 μL of centrifuged wine (12,000 x*g*, 4 °C, 10 min) were added to 100 μL of internal standard TSP solution at 2 mg/mL in deuterium oxide and combined with 250 μL of oxalate buffer [(HC_2_O_4_)^−^/(C_2_O_4_)^2−^ 0.11 M, pH 4.2] solution in D_2_O. After mixing, 600 μL were transferred in a 5-mm NMR tube for analysis. This procedure was performed in triplicate.

The ^1^H NMR spectra were automatically recorded at 298 K on a 600 MHz JEOL ECZ600R spectrometer (JEOL, Ltd., Japan). Deuterated water provided a field frequency lock, and TSP was used as chemical shift (*δ*) reference at ^1^H 0.00 ppm. The experiments were conducted at 25 °C, with a temperature delay of 120 s, non-spinning. Parameter sets included: a time domain of 32,768 complex data points (32*K*) using a 2796 Hz spectral width, 24 ppm sweep width, transmitter offset (x_offset = 4.664), 90° hard pulse (x_pulse = x90; x_atn = xatn) to be optimized by manual or automatic procedures, keeping pulse length < 10 μs, steady state (x_prescans = 4), number of transients (scans = 128), mixing time 0.010 s, a relaxation delay of 5 s, an acquisition time requirement of 2.2749 s and automatic receiver gain.

For ^1^H NMR analysis of alcoholic beverages such as wine, multiple solvent signal suppression is a major concern (Amargianitaki & Spyros, 2017). Indeed, conventional water suppression using presaturation for wine profiling lacks adequate sensitivity to detect minor compounds, such as aldehydes or phenolic compounds. So, to improve the spectral information, a specific shaped pulse sequence was applied during the relaxation delay to suppress the eight ^1^H NMR frequencies of water and ethanol (i.e., the OH singlet of water at 4.66 ppm, the CH_2_ quartet at 3.65 ppm and the CH_3_ triplet at 1.17 ppm of ethanol), by modifying the basic shape pulse obs_dante_presaturation reported in the JEOL ECZ600R database and previously applied for quantifying metabolites in grape juice (Musio et al., 2020). Also, the decoupling artifact removal of ^13^C satellite signals was performed using a pulse decoupling sequence during acquisition. An example of wine spectrum reporting signals for residual water, residual ethanol, elimination of ^13^C satellites and TSP chemical shift (*δ*) reference (at 0.00 ppm) can be found in the supplementary material (Fig. S1).

All the spectra were processed by Mnova software (release 6.0.2, Mestrelab Research, Spain) and manually phased; the baseline was corrected automatically. For quantitative analysis, an integration pattern was defined by manually choosing buckets between 0 and 10 ppm on all the acquired spectra in the overlapped form. This method enables the choice of sufficiently large buckets to compensate for the small chemical shift fluctuations in each spectrum and each bucket corresponds to a defined signal or a group of signals. This defined pattern identified eighty-four buckets for Amarone wine samples and was used for automatic integration, then refereed as percentage to TSP area and imported into an Excel file (Microsoft 365 MSO, version 2210, 64-bit) for further processing (described below). The assignment of ^1^H NMR signals was supported using data available in the literature (Musio et al., 2020) and the metabolomics data repository for NMR Metabolomics (bmrb.io).

### 2.4 Data analysis and chemometrics

LC-HRMS and ^1^H NMR data were formerly elaborated using Mass Profiler Professional software (version B.12.06; from Agilent Technologies). First, each feature's abundance was log2-transformed, normalized against the average percentage of dry matter measured for each withering time point (Table S1), and baseline-corrected against the median in the dataset. Unsupervised principal component analysis (PCA) was performed using the same software to explore metabolomic patterns naively. Then, multivariate analysis of metabolomics-based fingerprints was integrated further by applying supervised tools. As reported in a previous work (Zambianchi et al., 2023), Orthogonal Projections to Latent Structure Discriminant Analysis (OPLS-DA) was used as a supervised tool to consider only the Y-predictive variation and to eliminate the variation not directly correlated with Y in X (i.e., orthogonal signal correction). For this purpose, the raw dataset was exported in SIMCA 16 (Umetrics, Malmo, Sweden) for the supervised modelling, considering the four different withering time points as discriminant parameters. The OPLS-DA model validation parameters (goodness-of-fit R^2^Y together with goodness-of-prediction Q^2^Y) were recorded to evaluate the overall goodness of the prediction model. Regarding Q^2^Y prediction ability, a value >0.5 was adopted as an acceptability threshold to identify acceptable models. The OPLS-DA models were checked for outliers, and the permutation testing was performed to exclude overfitting (*N* = 100). Afterwards, the variable importance in projection (VIP) approach was exploited to highlight metabolites with the highest discriminating potential. This approach provides the contribution of each variable in the hyperspace separation of the OPLS-DA model, giving a specific VIP score. This factor is a weighted sum of the squared correlations between the OPLS-DA components and the original variables. Finally, a Fold-Change (FC) analysis was exploited to achieve information about the regulations of the VIP compounds, considering the different Amarone wines classified by the different withering points. Finally, the OPLS-DA supervised approach was repeated, according to the same procedure, but considering the type of yeast as the discriminant factor.

### 2.5 Multi-omics data integration and validation-prediction ability

The omics data integration analysis was performed to correlate the two omics datasets (^1^H NMR and LC–HRMS) and identify key variables during the integration process to produce a more consistent and accurate model able to characterize Amarone wine by using the R statistical computing program (version 4.1.3) using two different packages, namely omicade4 and mixOmics respectively. First, the multiple co-inertia analysis (MCIA) algorithm (Meng, Kuster, Culhane, & Gholami, 2014) is an unsupervised data exploration technique discovering co-relationships among multiple high-dimensional datasets. MCIA concurrently maps several datasets into a shared dimensional space using a covariance optimisation criterion. This transformation aligns different sets of features on a common scale, enabling the extraction of the most distinctive characteristics from each dataset. Afterwards, the multivariate supervised sparse Partial Least Squares Discriminant Analysis (sPLS-DA) was carried out on raw datasets with a sparse Generalized Canonical Correlation Analysis method (sGCCA). The sGCCA is a size reduction approach that enables feature selection, identification, and key predictors within the different omics datasets. Feature selection across omics levels is performed via ℓ1 penalization (Tibshirani, 1996) on the variable coefficient vector defining the linear combinations (González, Cao, Davis, & Déjean, 2012). Specifically, the two omics data sets were first normalized to the quantile and scaled to the median, then integrated with the sPLS-DA model. Three principal components were used to generate the integration model considering 100, 80 and 10 variables in the case of LC-HRMS data and 80, 6 and 50 variables in the case of ^1^H NMR data, respectively. The optimal number of components that achieved the best performance was chosen based on the overall error rate (BER). In contrast, the number of variables was determined by repeated and stratified cross-validation analysis (3-fold CV, repeated 50 times) to compare the performance of models built with different ℓ1 penalties.

## 3 Results and discussion

### 3.1 LC-HRMS

First, UHPLC-Orbitrap-HRMS analysis was used to investigate the chemical fingerprinting of Amarone wine samples based on two factors: i) withering time (four groups) and ii) yeast strain (two groups). Overall, considering the exclusive tandem-MS approach using QC samples allowed us to record an additional 323 compounds using the comprehensive database MoNA (Mass Bank of North America), provided by MS-Dial software. A detailed list of all the annotated compounds is reported in Table S2, along with their raw abundance and composite mass spectra. For the untargeted screening of wine samples, we used this MS/MS approach to improve the confidence of the identity confirmation process in non-target analysis.

After that, multivariate statistics based on unsupervised and supervised methods were carried out to investigate patterns according to their similarity and differences in metabolomic profiles. First, PCA was carried out to inspect the dispersion of each sample according to the sample classification, and the corresponding score plots of the first PCs (i.e., PC1 *vs* PC2) are reported as supplementary material (Fig. S2). From the withering time point of view, the score plot enhanced the impact of withering time on the studied wine samples. The separation occurs along the first principal component. The higher distance was detected between the first and the fourth levels of time points under investigation, with the more withered samples generally located at negative values on PC1. Therefore, we can postulate that the major changes in wine composition happened in the early stage of the withering process. Regarding the yeast strain's classification, no changes were detected among the samples, following a random distribution in the Euclidean space defined by the first two principal components. Therefore, LC-HRMS analysis, although no distinct separation occurred for wine fermented with different yeast strains, demonstrated a good ability to discriminate the chemical fingerprint of the withering time, involved in metabolic changes in grapevine berries, as previously reported (Zenoni et al., 2016).

**Fig. 1:**
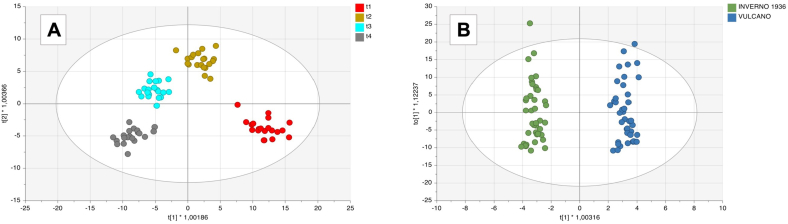
Supervised orthogonal projection to latent structures discriminant analysis (OPLS-DA) score plot considering the Amarone samples from different withering times (**A**) and different yeast types used in fermentation process using the LC-HRMS data (**B**).

After that, OPLS-DA models were produced considering both conditions to shed light on the specific contribution of the different metabolites for discrimination aims. For each model, overfitting was excluded by applying 100 random permutations, thus considering their cross-validation (*P* < 0.05). Then, variable importance in projection (VIP) combined with Fold-change analysis was used to select the most discriminant metabolites and to find information about their abundance in each factor. The outputs of these OPLS-DA models are shown in Fig. 1.

As observed, the supervised prediction model confirmed the outputs of unsupervised statistics applied to withering classification with excellent goodness of fitting (R^2^X = 0.442 and R^2^Y = 0.936) and prediction ability (Q^2^ = 0.877). A comprehensive list containing all the discriminant metabolites for withering time conditions (having a VIP score > 1) is reported as supplementary material (Table S3). Furthermore, to shed light on the marker compounds better responsible for the metabolomic differences at the more distant time points (i.e., t4 *vs* t1), a fold-change (FC) analysis (FC > 1.2; P < 0.05) on the VIP compounds (grouped in chemical classes) was carried out. Overall, as we can observe in Table 1, a high number of classes could be outlined from the LC-HRMS data, with amino acids (14 compounds), lipids (14 compounds), flavonoids (7 compounds), and carbonyl compounds (4 compounds) being the most represented classes among the discriminant compounds.

**Table 1:** Comprehensive summary of the most discriminant compound classes calculated for the VIP markers of OPLS-DA models detected by LC-HRMS analysis built considering the withering time (t1-t4) as a factor parameter. Specifically, for each class annotated, the average VIP score together with the most discriminant compound and Log Fold-Change (FC) value based on the “t4 *vs* t1” comparison is provided.

**Chemical Class**	**Number of VIP metabolites**	**Most discriminant metabolites**	**Average VIP scores**	**Average Log FC scores (t4 *vs* t1)**
Amino acids and derivatives	14	L-Lysopine (VIP score = 1.550)	1.169	0.750
Benzene and substituted derivatives	1	4-O-Methylgallic acid 3-O-sulphate	1.084	−1.650
Carbohydrates and carbohydrate conjugates	1	O-6-deoxy-a-L-galactopyranosyl-(1- > 2)-O-b-D-galactopyranosyl-(1- > 4)-2-(acetylamino)-1,5-anhydro-2-deoxy-D-arabino-Hex-1-enitol (VIP score = 1.252)	1.252	2.670
Carbonyl compounds	4	4-(2-Amino-3-hydroxyphenyl)-2,4-dioxobutanoic acid (VIP score = 1.405)	1.255	−1.530
Carnitines and derivatives	2	l-Carnitine (VIP score = 1.632)	1.328	1.700
Carotenes	1	7,8-Dihydrolycopene	1.739	3.530
Cinnamic acids and derivatives	1	Fertaric acid	1.000	−0.110
Dipeptides	2	gamma-Glutamylmethionine (VIP score = 1.141)	1.080	−0.550
Diterpenoids	2	Cincassiol B (VIP score = 1.331)	1.311	0.690
Flavonoids	7	Cirsiliol (VIP score = 1.369)	1.172	0.300
Glycerolipids and derivatives	3	LysoPA(18:0/0:0) (VIP score = 1.284)	1.237	2.530
Indoles and derivatives	1	Methyl oxindole-3-acetate	1.299	1.180
Keto acids and derivatives	2	2-Keto-glutaramic acid (VIP score = 1.079)	1.078	−0.700
Lineolic acids and derivatives	1	Stearidonic acid	1.062	−6.190
Lipids and derivatives	14	PA (18:1(9Z)/18:1(11Z)) (VIP score = 1.759)	1.342	0.670
Monoterpenoids	2	Valtrate (VIP score = 1.283)	1.159	3.990
Phenolic glycosides	2	3-Glucosyl-2,3′,4,4′,6-pentahydroxybenzophenone (VIP score = 1.373)	1.209	−1.050
Purine nucleosides	2	Adenylsuccinic acid (VIP score = 1.438)	1.306	−1.120
Pyridines and derivatives	1	3-Hexylpyridine	1.529	−5.480
Pyrimidine nucleosides	2	dTDP-d-glucose (VIP score = 1.246)	1.150	0.050
Sesquiterpenoids	3	Melleolide C (VIP score = 1.260)	1.130	1.320
Stilbenes	1	Resveratrol	1.345	3.300
Triterpenoids	1	Ganoderic acid Mj	1.110	2.000
Other	13	7-Hydroxy-2-methyl-4-oxo-4H-1-benzopyran-5-acetic acid (VIP score = 1.289)	1.159	0.964

Abbreviations: PA: phosphatidic acid; LysoPA: lysophosphatidic acid; dTDP-d-glucose: Deoxythymidine diphosphate-d-glucose

In more detail, the L-Lysopine (VIP score = 1.55), the phosphatidic acid PA (18:1(9Z)/18:1(11Z)) (VIP score = 1.759), Cirsiliol (VIP score = 1.369), and 4-(2-Amino-3-hydroxyphenyl)-2,4-dioxobutanoic acid (VIP score = 1.405) were found to possess the highest discrimination potential, respectively. In particular, amino acid compounds showed a mild change during the overall withering time conditions under investigation, except for glutathione, which showed an early increase up to 8.82 Log FC in t1 when compared to t4. In this scenario, a significant difference in the accumulation of isoleucine, asparagine, and *N*-acetylglutamine occurred among the two extreme withering stages (i.e., t4 *vs* t1). Also, most of the lipids highlighted were found to be associated with a general up-regulation, describing mainly wines produced with more withered grapes. In particular, it is well established that withering represents a complex process characterised by chemical and biochemical reactions that affect lipolytic activities in wine. As confirmed by Mahanta, Tamuli, and Bhuyan (1993), a continuous increase in free fatty acid content could be observed during the evolution of withering. Interestingly, among lipid compounds, we also found a derivative of 3-methylbutanoate, which has already been detected in red wine as one of the main fatty alcohol esters strongly influencing wine aroma (Pavez, Steinhaus, Casaubon, Schieberle, & Agosin, 2015). Regarding the other classes of compounds, a substantial increase of resveratrol was detected from the initial time point, suggesting improved nutritional status of raisin grape during the period of withering (Log FC value = 3.30). As reported by Versari, Parpinello, Battista Tornielli, Ferrarini, & Giulivo, 2001, the biosynthesis of stilbenes is promoted by several factors, like abiotic stress and cell dehydration, and the combined effect of time and temperature seems to play an essential role as a stress factor in resveratrol accumulation during post-harvest of Corvina grape berry used in Amarone production.

Surprisingly, the OPLS-DA model originated considering the two different fermentation processes of winemaking showed interesting cross-validation parameters (R^2^X = 0.4, R^2^Y = 0.979, and Q^2^ = 0.785). The introduction of an orthogonal signal correction allowed for a clear separation between the two observation groups along the orthogonal latent vector (i.e., Vulcano *vs* Inverno 1936). Regarding the most discriminant metabolites obtained by the OPLS-DA model by yeast, 37 compounds were particularly able to explain the differences related to the inoculation of different yeast species exploiting the wine fermentation. These marker compounds are reported in Table S4, organized in classes according to the database FooDB (as previously described). In our experimental conditions, the most discriminant metabolites between the two yeast strains belonged to amino acids, accounting for 43% of the VIP compounds (VIP score > 1). As can be observed, amino acids showed an average down accumulation for the comparison “Vulcano *vs* Inverno 1936” with galactosyl 4-hydroxyproline (VIP score = 2.384), ornithine (VIP score = 2.147), L-glutamic acid (VIP score = 1.686), l-serine (VIP score = 1.557) showing the highest VIP scores. Succinic acid was another compound deeply affected by fermentation; our findings revealed its presence with a moderate discriminating impact in the different wine samples according to the yeast used (VIP score = 1.216). Succinic acid, an important organic acid in wine, plays a vital role in imparting microbial stability to wines through its influence on acidity and pH (Torres-Guardado, Rozès, Esteve-Zarzoso, Reguant, & Bordons, 2022). The quantity of succinic acid produced during alcoholic fermentation depends on factors such as yeast strain, fermentation temperature, and the chemical composition of the wine (Aplin, Paup, Ross, & Edwards, 2021). To date, data about the variation of succinic acid for grape wine fermented with *S. bayanus* compared to *S. cerevisiae* yeast are not available, so further studies must be performed on this topic. Finally, no clear explanation of the other discriminant compounds' involvement in the wine fermentation process has been found yet.

### 3.2 ^1^H NMR

In the second part of this work, we tested the ability of untargeted metabolomics based on ^1^H NMR to comprehensively screen and profile low-molecular-weight compounds able to define the different Amarone wine samples. Overall, 83 integrating areas were obtained from all the 80 ^1^H NMR acquired spectra, including TSP area, but excluding from the bucketing the spectral regions of residual water and ethanol. Among these, 42 signals (corresponding to 31 metabolites or classes of metabolites) were assigned. The obtained dataset (normalized TSP areas, ^1^H chemical shift (*δ*, ppm range) and proton assignment) is reported in Table S5.

**Fig. 2:**
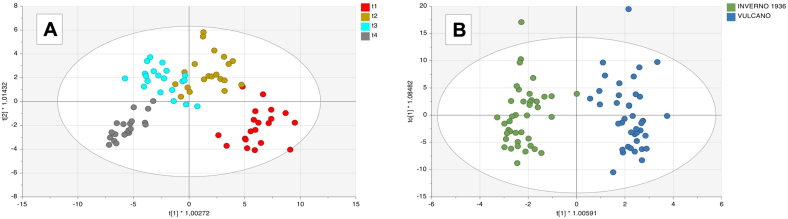
Supervised orthogonal projection to latent structures discriminant analysis (OPLS-DA) score plot considering the Amarone samples from different withering times (**A**) and different yeast types used in fermentation process using the ^1^H NMR data (**B**).

As the next step, PCA was performed as a preliminary step for visually exploring patterns within the ^1^H NMR dataset. As shown in Fig. S3, the 83 contributions tend to separate wine samples according to the different withering time points, obtaining a very interesting separation along the first principal component, accounting for 40.25% of the total variability. Amarone wines originating from grapes with a prolonged withering were in the negative region of PC1, whereas the wine samples, classified as “t1”, were in the positive region of PC1. Regarding the different yeasts used during wine fermentation, no clear separation trends were observed between the wine samples, indicating that the ^1^H NMR dataset is mainly influenced by the withering time variability. As the next evaluation, for further classification of the ^1^H NMR dataset, OPLS-DA models were built based on the results above. So, withering time (t4 *vs* t1) and yeast strains (Vulcano *vs* Inverno 1936) were tested again as class discriminant parameters for wine sample classification. The two discriminant models and VIP scores of discriminant variables were calculated, and the corresponding score plot is represented in Fig. 2.

In both score plots, samples tend to separate between groups defined by the selected factors. As can be observed from Fig. 2, the supervised prediction model built for the withering time class allowed confirming the outputs of unsupervised statistics with the first latent vector, discriminating between the t1 withered wine samples and the other withering stages. Concerning yeast strains, we found an evident separation trend between the two different yeasts, thus confirming the discriminating power of this supervised approach to distinguish wines fermented with different yeast strain types. In this regard, the model cross-validation parameters of both OPLS-DA models were satisfactory, being R^2^X = 0.711, R^2^Y = 0.776 and Q^2^ = 0.674 for the withering time condition, and R^2^X = 0.617 R^2^Y = 0.921 and Q^2^ = 0.844 for yeast strain classification. Again, 100 random permutations and the cross-validation (CV)-ANOVA (considering a *P* < 0.05) were performed to prevent model overfitting.

Then, we extrapolated the most discriminant compounds from the ^1^H NMR dataset, allowing sample discrimination as a function of i) withering time and ii) yeast strain, according to the VIP selection method (VIP score > 1). Regarding the withering time condition, the 31 contributions characterizing the “withering time” can be found in Table 2.

**Table 2:** Comprehensive summary of the most discriminant compound classes calculated for the VIP markers of OPLS-DA models detected by ^1^H NMR analysis built considering the withering time (t1-t4) as a factor parameter. Specifically, for each VIP compound annotated, VIP score together with Log Fold-Change (FC) value based on the “t4 *vs* t1” comparison is provided.

**Class**	**VIP metabolites**	**δ (ppm)**	**VIP scores**	**Log FC ([t4] *vs* [t1]**	**Regulation [t4] *vs* [t1]**
Alcohols and polyols	methanol	3.35–3.34	1.134 ± 0.365	−0.31	down
	shikimic acid	6.81–6.80	1.054 ± 0.258	0.29	up
Alkaloids and derivatives	trigonellin	8.10–8.04	1.164 ± 0.061	0.49	up
	trigonellin	8.85–8.80	1.101 ± 0.058	0.24	up
	trigonellin	9.13–9.10	1.069 ± 0.076	0.15	up
Amino acids, peptides, and analogues	tyrosine	7.20–7.16	1.519 ± 0.431	−0.65	down
	tyrosine	6.86–6.83	1.484 ± 0.446	−0.8	down
	threonine	1.32–1.31	1.379 ± 0.639	−0.92	down
Carbohydrates and carbohydrate conjugates	mannitol	3.79–3.74	1.125 ± 0.261	0.03	up
	monosaccharide	4.58–4.54	1.092 ± 0.196	0.37	up
	disaccharide (sucrose)	5.44–5.42	1.049 ± 0.088	0.35	up
	fructose	4.01–3.98	1.021 ± 0.741	0.58	up
	alpha-glucose	5.22–5.20	1.007 ± 0.564	0.64	up
Carbonyl compounds	acetoin	2.22–2.21	1.083 ± 0.792	−0.55	down
	ethanal	2.23–2.22	1.406 ± 0.64	−1.36	down
Carboxylic acids and derivatives	acetic acid	2.07–2.03	1.176 ± 0.203	0.8	up
Cinnamic acid and derivatives	caffeic acid (2)	6.53–6.47	1.106 ± 0.285	0.76	up
Not assigned	Metabolite 40	9.68–9.64	1.394 ± 0.641	−1.8	down
	Metabolite 39	9.13–9.10	1.16 ± 0.13	1.1	up
	Metabolite 10	1.71–1.68	1.143 ± 0.102	−0.08	down
	Metabolite 22	5.30–5.28	1.134 ± 0.198	0.26	up
	Metabolite 28	6.90–6.87	1.099 ± 0.147	0.27	up
	Metabolite 38	8.40–8.30	1.09 ± 0.245	1.09	up
	Metabolite 32	7.43–7.40	1.051 ± 0.13	0.54	up
	Metabolite 15	3.14–3.08	1.044 ± 0.194	0.57	up
	signals overlapping	2.53–2.38	1.043 ± 0.145	0.01	up
	Metabolite 21	5.25–5.22	1.041 ± 0.426	−0.36	down
	Metabolite 14	2.36–2.35	1.027 ± 0.373	0.2	up
	Metabolite 35	7.99–7.96	1.022 ± 0.549	−0.88	down
	Metabolite7	1.29–1.26	1.018 ± 0.293	−0.09	down
	Metabolite 18	3.89–3.86	1.012 ± 0.636	0.64	up

As can be observed, we confirmed that carbohydrates were enriched in wine samples with a longer withering period, with different regions associated with monosaccharide compounds showing an overall up-accumulation trend. Our data revealed the presence of shikimic acid as one of the most discriminant metabolites, associated with a relatively high VIP score value (VIP score = 1.054) and characterizing mainly the t4 wine samples (Log FC = 0.29). This compound is one of the precursors for aromatic amino acids, like phenylalanine and tyrosine. Tyrosine showed the highest VIP score with two different contributions at 6.86–6.83 and 7.20–7.16 ppm, respectively. It was generally recognized as a polyphenol precursor during red wine withering (Scalzini, Giacosa, Río Segade, Paissoni, & Rolle, 2021). Furthermore, grape dehydration had a particular influence on the increase of acetic acid and phenol compounds, like caffeic acid, as the consequence of gene expression in the skin of winegrape berries subjected to partial postharvest dehydration (Bonghi et al., 2012). Among wine compounds having the strongest discrimination potential (Table 2), 13 metabolites were unsuccessfully assigned to any specific molecule due to overlapping and low intensities of signals and the lack of reference materials.

Otherwise, the results obtained by the OPLS-DA model built by yeast type revealed the presence of 16 contributions in defining the different fermentations carried out to produce Amarone wine. The comprehensive list of the VIP contributions is reported in Table S6. In particular, five features were linked to specific identifications, namely lactic acid, isopentanol, arginine, isobutanol, and phenethyl alcohol, which showed different accumulation trends in wine subjected to different fermentation strategies. Specifically, Amarone wines produced using *S. bayanus* as yeast strain (i.e., Vulcano) were found to be enriched in isopentanol, isobutanol, and lactic acid when compared to wines obtained using *S. cerevisiae* for wine fermentation (i.e., Inverno 1936). Overall, inspecting the diversity of secondary metabolites originating from the different fermentation processes demonstrated the crucial impact of selecting the right yeast strain, as it significantly influences the final characteristics and flavour profile of the wine (Tosi, Azzolini, Guzzo, & Zapparoli, 2009). Based on this evidence, ^1^H NMR untargeted metabolomics confirmed its potential in investigating the potential effect of specific yeast types on the chemical fingerprint of Amarone wine samples.

### 3.3 Multi-omics data integration

Finally, a holistic approach was considered to interpret and integrate high-dimensional data. In this regard, we combined the two untargeted metabolomics approaches adopted for the overall fingerprint of Amarone wines, both of withering times (i.e., t1-t4) and yeast strains (Vulcano *vs* Inverno 1936) point of view, by using an unsupervised MCIA method. This approach was used to jointly analyze the two datasets, and the graphical outputs of this analysis are shown in Fig. S4. As we observed, the first two axes could highlight differences among the different withering times, with the second component having the highest discrimination potential (Fig. S4A). Otherwise, as previously reported, no significant peculiarities were highlighted with the yeast strain classification, confirming the output of unsupervised statistics carried out with both datasets (Fig. S4B). Furthermore, by inspecting the pseudo-eigenvalue space given by the two datasets (Fig. S4E), we observed that the contribution and the accordance of the two datasets to the model are low, with an overall scarce correlation RV-score of 16.4%. This finding suggested the complementarity of the information belonged to the two datasets; based on that, the contribution of each group of wine metabolites was next investigated according to supervised sparse PLS-DA. The sparse version of the PLS-DA method incorporated a Lasso penalty to improve classification accuracy, and it is usually employed to manage and analyze the integration of large omics, using the sample vector as an anchor between datasets, with a special focus on variable selection (Lê Cao, Rossouw, Robert-Granié, & Besse, 2008). The key variables selected were considered highly correlated multi-omics signatures able to discriminate and explain the outcome of interest according to the metadata. For this purpose, we first selected the optimum parameters to build the sPLS-DA model for withering time points (Fig. S5) and for yeast strains (Fig. S6) of Amarone wine production by using tuning analysis that calculates the cumulative amount of explained variance for a wide number of principal components, then repeated cross-validation analysis was performed to assess their performance.

Notably, the number of sparse components and features important for integration model building were selected according to tuning plots obtained by the two datasets, shown in Fig. S5 (c-d) for withering times and in Fig. S6 (c-d) for yeast strain models. According to our results, three sparse components were chosen for both models, achieving the lowest classification error rate, as determined with a one-sided *t*-test. Estimating the classification error rate concerning the variables' number, one component was carried out at a time to better predict the optimal number of variables with higher discrimination capacity. The performance of sPLS-DA models generated using selected parameters was investigated with repeated cross-validation processes to assess their ability to correctly classify ‘new’ samples based on withering times (Fig. S5a and S5b) or different yeast strains (Fig. S6a and S6b). Generally, the error rate decreases and reaches a minimum at three sparse components with Mahalanobis distance for both models. According to optimum parameters selected previously, the two datasets were integrated using a sparse PLS-DA model, and the contribution of each variable chosen to each sparse component was highlighted for each dataset using a correlation circle plot, shown in Fig. S5e (for withering times) and in Fig. S6e (for yeast strains). Overall, the first two components produced correlated cluster points among the sparse components, reporting positive and negative correlations between features selected from the two datasets. Specifically, the features selected among two datasets to build withering times models resulted in highly positive and negative correlations along component 1 (Fig. S5e). The correlation structure is less evident in component 2, but we observe some key selected features that significantly contribute to this component. The selected features used to build the yeast strains model were reported as highly correlated along component 2, showing clear positive and negative cluster points in that dimension (Fig. S6e).

#### 3.3.1 Discrimination models by withering

**Fig. 3:**
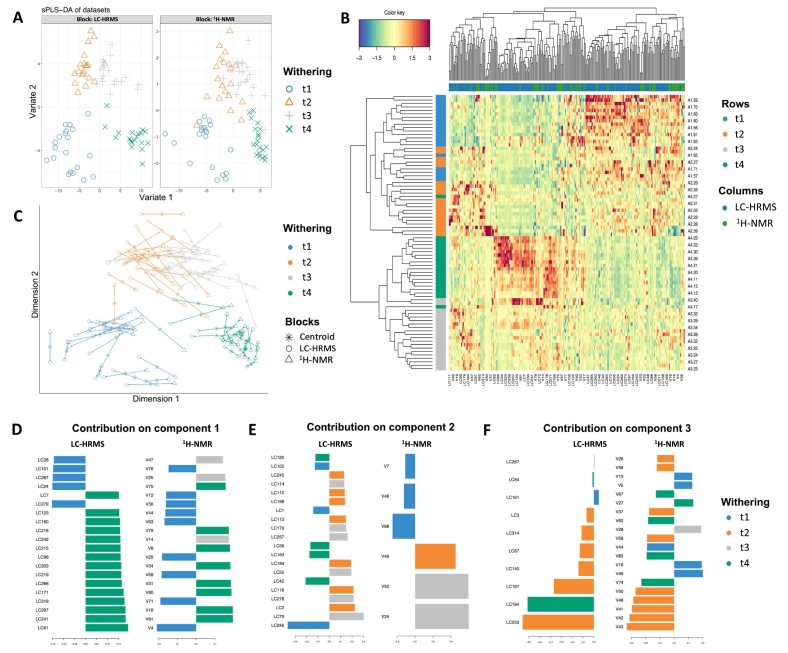
sPLS-DA model performed using LC-HRMS and ^1^H NMR datasets for withering time effect. (**A**): Samples plots of the first two components for each dataset, which are coloured based on withering time points. (**B**) Heatmap of the selected features by sPLS-DA on the withering times across the three considered components, with samples in rows coloured according to their withering times. (**C**): Arrow plot from integrated sPLS-DA projected into the space spanned by the first two components for each dataset, then overlaid across datasets. (**D—F**) Loading plots of the selected features by sPLS-DA for each of the three sPLS-DA components considered: (**D**) component 1; (**E**) component 2; (**F**) component 3. Features are ranked according to their loading weights represented as a bar plot. Colours indicate the class for which a particular compound is maximally present.

The overall outcome of the sPLS-DA discrimination model built to discriminate withering times is reported in Fig. 3, in which panel 3 A shows the sample plot of each sample into the space spanned by the components from each dataset involved in the analysis.

The first component of the sPLS-DA model discriminated withering t1-t2 from t3-t4 using 100 and 80 features as LC-HRMS and ^1^H NMR datasets, respectively. The second and third components further discriminated all withering times based on overall 90 and 56 features extrapolated from the two datasets, respectively. Specifically, the features with negative weight were highly present in withering times t1 and t4, while features with positive weight were present in t2 and t3. This outcome was also confirmed by the arrow plot reported in Fig. 3C. The samples belonging to the two datasets were grouped according to withering times, as reported in the sample plot. Interestingly, the integration model was characterised by a low distance between datasets, highlighted by the length of arrows connecting the same sample under the two dataset's dimensions, and the start of the arrow is their centroid. The hierarchical clustering analysis built with Euclidean distance with the Complete agglomeration method was carried out using a heatmap based on the modulation of all features selected across the three sparse components with respect to each sample (Fig. 3B). The variables selected produced two main clusters according to withering times, and features selected across the two datasets reported similar up or down modulation in the clustering group. Finally, loading plots of the first 20 variables on each sparse component are represented in Fig. 3d-f as the first, second, and third components, respectively, and a complete list of features with loading weights was reported in Table S7. The selected features from the two datasets were accessed for their overall Pearson's correlation, reporting as *r* = 0.94, *r* = 0.89, and r = 0.89, as components 1, 2, and 3, respectively. The different colours of each bar are determined based on the maximal contribution of the selected feature to the class discrimination. Considering the features contributing to component 1, they mainly belonged to the group of withering t1 and t4, confirming a discrimination capacity of that component to t1 and t4. The most correlated features between the two datasets were associated with withering t4, characterised by amino acids and their derivatives, lipids, aromatic amines, alcohols, and terpenoids. At the same time, t1 was mostly enriched of carbohydrates, flavonoids, organic acids, and carbonyl compounds. The second and third components of sPLS-DA were mainly involved in discriminating t2-t3 and t1-t4, confirming the amino acids and lipids accumulation during the withering process. The sPLS-DA model was validated using repeated cross-validation analysis (3-fold with 50 repeats), choosing Mahalanobis distance. The classification error rate produced an overall error rate (ER) equal to 7.52%, resulting in a solid model (Fig. S7a). Moreover, ROC (Receiver Operating Characteristics) analysis was performed to investigate the classification potential of metabolites selected from both the LC-HRMS (Fig. S7b) and ^1^H NMR blocks (Fig. S7c). Both models reported overall high AUC (Area Under the Curve) values, obtained using one-*vs*-all comparisons, indicating a high true positive rate (sensitivity on the y-axis) and a high true negative rate (or low 100 - specificity on the x-axis), with an AUC close to 1. In this case, we reported a high classification on withering t2 (AUC = 0.9967) and t4 (AUC = 0.9675) for the LC-HRMS dataset and withering t4 (AUC = 0.9933) and t3 (AUC = 0.9675) for ^1^H NMR dataset, considering all the three components. All the AUC values obtained from the one-*vs*-all comparisons were statistically significant (*P* < 0.05), assessed using the Wilcoxon test *p*-value performed per component.

In general, it is known that the post-harvest withering process of grapes lasts up to three months and alters the structure of berries due to the loss of water, and partially dried fruit conserves and releases desirable attributes during the wine-making process (Bellincontro, de Santis, Botondi, Villa, & Mencarelli, 2004). In particular, we observed a mild change in amino acid compounds during the overall withering time. In this scenario, a significant difference in the accumulation of isoleucine, alanine, lysine, arginine, glutamine, and several dipeptides occurred during the withering process. The increase in branched-chain amino acids like isoleucine or peptides has been previously suggested to contribute to Amarone wine's specific aroma and water-deficit-stressed mature berries (di Carli et al., 2011). This trend could be explained by the degradation of large protein molecules due to the water deficiency, facilitating the proteases and peptidase enzymes released from damaged cells. Moreover, we observed the accumulation of monosaccharides and fructose during the withering process. It is established that elevated sugar levels in berry tissues can activate the shikimic acid pathway, thereby increasing significant classes of non-volatile metabolites (Sanmartin et al., 2021). Our data revealed the presence of shikimic acid, which resulted as one of the discriminant metabolites in the withering t4 group and was associated as one of the precursors for the aromatic amino acids, like phenylalanine and tyrosine. Interestingly, the latest two amino acids were highly discriminant in the withering t4 and recognized as a direct precursor of non-flavonoid phenolics implied during withering in red wine (Scalzini et al., 2021). Indeed, the withering condition of grapes induces specific changes in the accumulation of flavonoids and polyphenols (Brillante, Vedova, Maoz, Flamini, & Tomasi, 2018). Our findings show that some flavonoids like myricetin, astilbin, and quercetin 3-gluco-xyloside decreased with the grape dehydration process, while kaempferol 3-sophorotrioside, 7,4′-dihydroxyflavone, and cyanidin 3-(diferuloylsophoroside) 5-glucoside resulted specifically increased at t4.

#### 3.3.2 Discrimination models by yeast

**Fig. 4:**
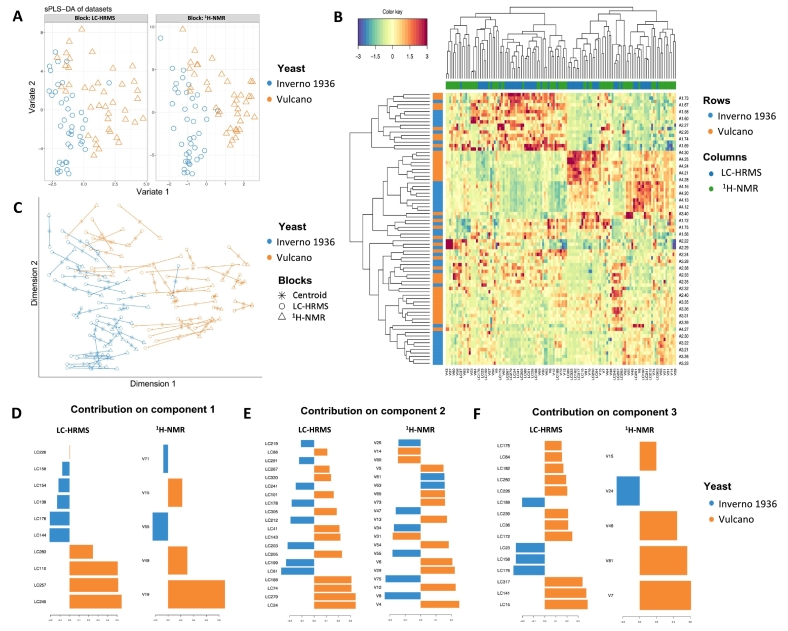
sPLS-DA model performed using LC-HRMS and ^1^H NMR datasets for yeast strain effect on wine fermentation. (**A**): Samples plots of the first two components for each dataset, which are coloured based on yeast type. (**B**) Heatmap of the selected features by sPLS-DA on the yeast factor across the three considered components, with samples in rows coloured according to the yeast strain used for Amarone wine fermentation. (**C**): Arrow plot from integrated sPLS-DA projected into the space spanned by the first two components for each dataset then overlaid across datasets. (**D—F**) Loading plots of the selected features by sPLS-DA for each of the three sPLS-DA components considered: (**D**) component 1; (**E**) component 2; (**F**) component 3. Features are ranked according to their loading weights represented as a bar plot. Colours indicate the class for which a particular compound is maximally present.

The integrated sPLS-DA model made for the discrimination of the two yeast strains used in the Amarone fermentation process, namely Inverno 1936 (*S. cerevisiae*) and Vulcano (*S. cerevisiae* var. *bayanus*), is reported in Fig. 4a.

The resulting sample plot shows a clear classification capacity on the discrimination of the two yeast strains used for the Amarone fermentation by the contribution of component 1. The outcome was also confirmed using an arrow plot (Fig. 4c), displaying a separated distribution of the samples in the sparse dimensions in accordance with the yeast strain used. Moreover, the distance between the two datasets was distinctly short (see the length of the arrows). The HCA obtained using selected features across the three components of the two datasets blandly produced clear clusters. This behaviour could be explained by the fact that the withering time factor had more impact on the wine fermentation process compared to the employment of yeast strains. Despite the difficulty of the sPLS-DA model in discriminating between the two yeasts, the model was characterised by good classification performance, reporting an overall error rate of 10.45% determined by 3-fold cross-validation analysis (50 repeats; Fig. S8a). Accordingly, the variables selected across the components provided a distinctive profile of the critical metabolites produced by the two yeast strains (Fig. 4d-f). The complete list is reported in Table S8, including compound name, compound classification, and discrimination weight. The wine fermented with the two yeasts reported several common metabolites, including alcohols, aromatic amines, amino acids, and simple carbohydrates. However, several other metabolites discriminated between Vulcano *vs* Inverno 1936, highlighting the enrichment of flavonoids, lipids, and organic acids. The discriminant variables were validated using ROC analysis for the LC-HRMS (Fig. S8b) and ^1^H NMR blocks (Fig. S8c). Both models highlighted great and statistically significant AUC values (*P* < 0.05), obtained by Vulcano *vs* Inverno 1936 comparison, as 0.9488 and 0.9494 for LC-HRMS and ^1^H NMR, respectively, considering all three components.

Overall, amino acids have already been detected as possible discriminant biomarkers during alcoholic fermentation. The amino acid content of must before fermentation may affect the production of many volatile compounds that contribute to wine flavour and serve as direct metabolic precursors for synthesizing higher alcohols, short- to medium-chain fatty acids, and their ethyl ester or acetate ester derivatives. However, some differences in amino acid composition based on yeast strains have been observed, including the accumulation of arginine, tyrosine, ornithine, and isoleucyl-tryptophan, mainly related to *S. bayanus* yeasts' fermentation. Furthermore, considering alcohol production, the integrated sPLS-DA model highlighted a clear difference between the two yeast strains. Specifically, we observed a higher production of isopentanol in wine fermented with *S. bayanus* yeast (according to our findings in the ^1^H NMR OPLS-DA model built for yeast), while 2,3-butanediol showed a stronger association with *S. cerevisiae* yeasts. In particular, as depicted by Sottil et al. (2019), isomers of 2,3-butanediol directly contributed to the overall complexity of the wine sensory profile, with fatty and fruity tastes. Also, the integrated model highlighted several flavonoids as discriminant biomarkers of wine fermentation conducted with *S. bayanus* and *S. cerevisiae* yeasts, detecting three flavonoids characterised by high discrimination weights, such as 3,7-dimethylquercetin, epicatechin, and sativan. Finally, among the VIP metabolites, we found caproic acid as a lipid-derived discriminant compound detected along the first latent variable, showing a high loading score value (i.e., 0.51). As stated by Liu et al. (2022), a stronger correlation with high levels of this compound was observed in wines fermented with Saccharomyces-based yeasts.

## 4 Conclusion

Our study demonstrated the potential of multi-omics data fusion as a powerful tool for characterizing Amarone wine. This strategy, exploiting both unsupervised and supervised methods, demonstrated a high capability to manage the integration of large omics datasets and identify key metabolites crucial for discriminating wine samples according to their unique characteristics. Specifically, by integrating untargeted metabolomics data from LC-HRMS and ^1^H NMR techniques, we achieved a much deeper insight into the wine profile. The limited correlation (RV-score = 16.4%) between the datasets highlighted the complementarity of the information belonged to the two datasets. Moreover, our supervised statistical approaches based on sparse PLS-DA classified wine samples according to withering time and yeast strain conditions, providing a more comprehensive characterization of the wine metabolome when compared to individual approaches. Notably, we found that the withering process had the most significant discriminative impact on our data, resulting in a remarkably low error rate (7.52%) in sample classification (whereas an error rate of 10.45% for yeast strain classification). This observation was further supported by significant variations in the accumulation of amino acids, monosaccharides, and polyphenolic compounds throughout the withering process. To conclude, this multi-omics data fusion approach offers valuable insights for both researchers and wine industries, providing a robust framework for characterizing and assessing the quality of this renowned wine variety.

## CRediT authorship contribution statement

**Pier Paolo Becchi:** Writing – review & editing, Writing – original draft, Investigation, Formal analysis. **Veronica Lolli:** Writing – review & editing, Writing – original draft, Investigation, Formal analysis. **Leilei Zhang:** Writing – review & editing, Data curation. **Francesco Pavanello:** Funding acquisition, Conceptualization. **Augusta Caligiani:** Writing – review & editing, Supervision, Methodology, Conceptualization. **Luigi Lucini:** Writing – review & editing, Visualization, Methodology, Conceptualization.

## Declaration of competing interest

The authors declare that they have no known competing financial interests or personal relationship that could have appeared to influence the work reported in this paper.

## Data Availability

No data was used for the research described in the article.

## Appendix A Supplementary data

Supplementary material 1 Table S1. Amarone wine sample legend and total dry extract (g/l). Supplementary material 1

Supplementary material 2 Table S2. metabolomics dataset resulting from the LC-HRMS analysis (MS-DIAL software). Supplementary material 2

Supplementary material 3 Table S3. VIP markers of LC-HRMS analysis related to withering time class of information. Supplementary material 3

Supplementary material 4 Table S4. VIP markers of LC-HRMS analysis related to the different yeast strains used for wine fermentation. Supplementary material 4

Supplementary material 5 Table S5. ^1^H NMR metabolomic dataset, chemical shift values (ppm range), proton assignments (ID) and normalized TSP areas in analysed wine samples. Supplementary material 5

Supplementary material 6 Table S6. VIP markers of 1H-NMR analysis related to the different yeast strains used for wine fermentation. Supplementary material 6

Supplementary material 7 Table S7. Complete list of features with loading weights of sPLS-DA analysis for withering time. Supplementary material 7

Supplementary material 8 Table S8. Complete list of features with loading weights of sPLS-DA analysis based on yeast strains. Supplementary material 8
