# Supplementary material for: Integration of LC-HRMS and 1H NMR metabolomics data fusion approaches for classification of Amarone wine based on withering time and yeast strain
Source: Food Chem X. 2024 Jul 2;23:101607. doi: 10.1016/j.fochx.2024.101607 (PMC11279979; doi:10.1016/j.fochx.2024.101607)
Supplement: Supplementary material 1 — Table S1. Amarone wine sample legend and total dry extract (g/l). [file mmc1.docx]

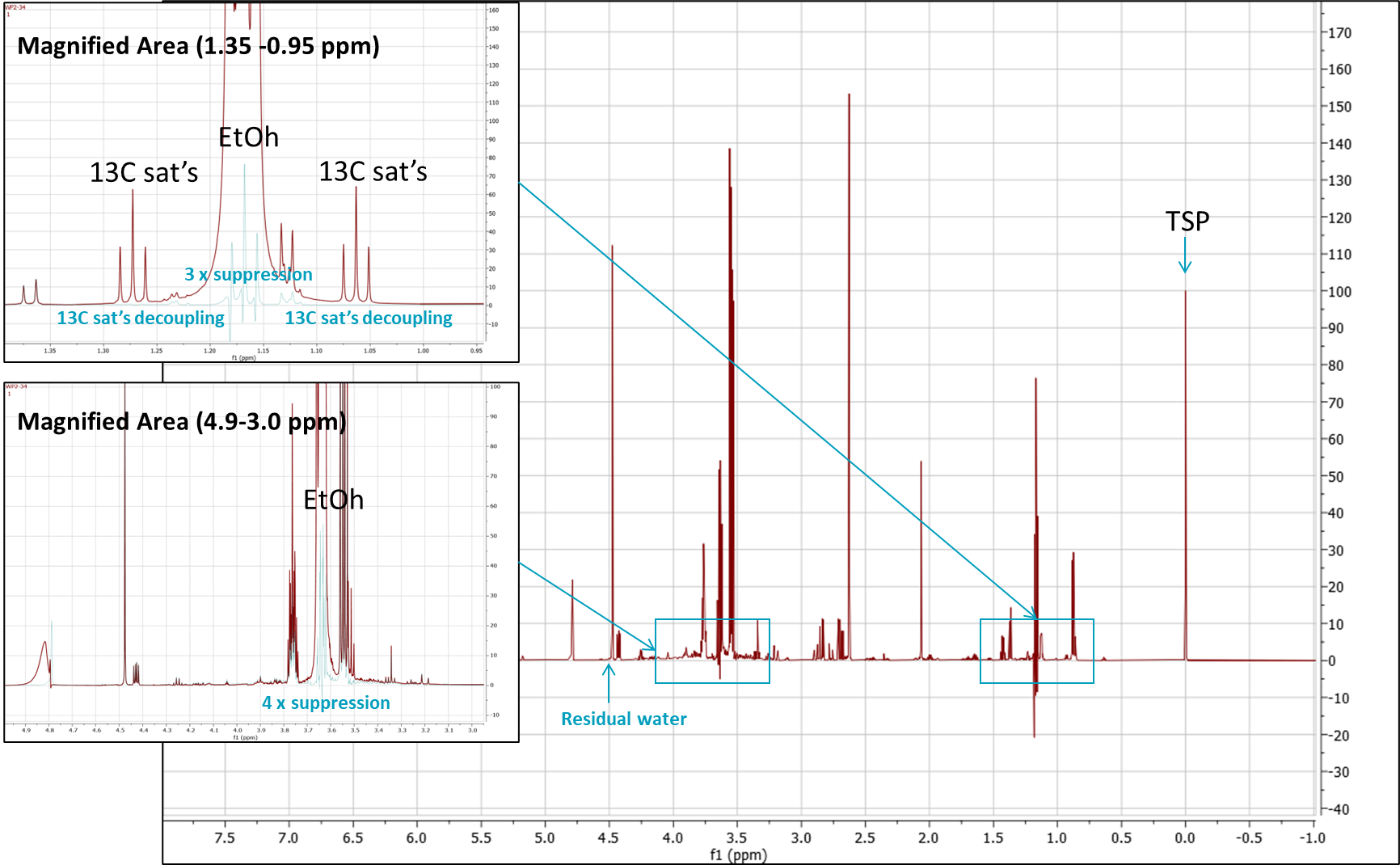


**Figure S1.** ^1^H-NMR wine spectrum reporting signals for residual water, residual ethanol, elimination of ^13^C satellites and TSP chemical shift (δ) reference (at 0.00 ppm).


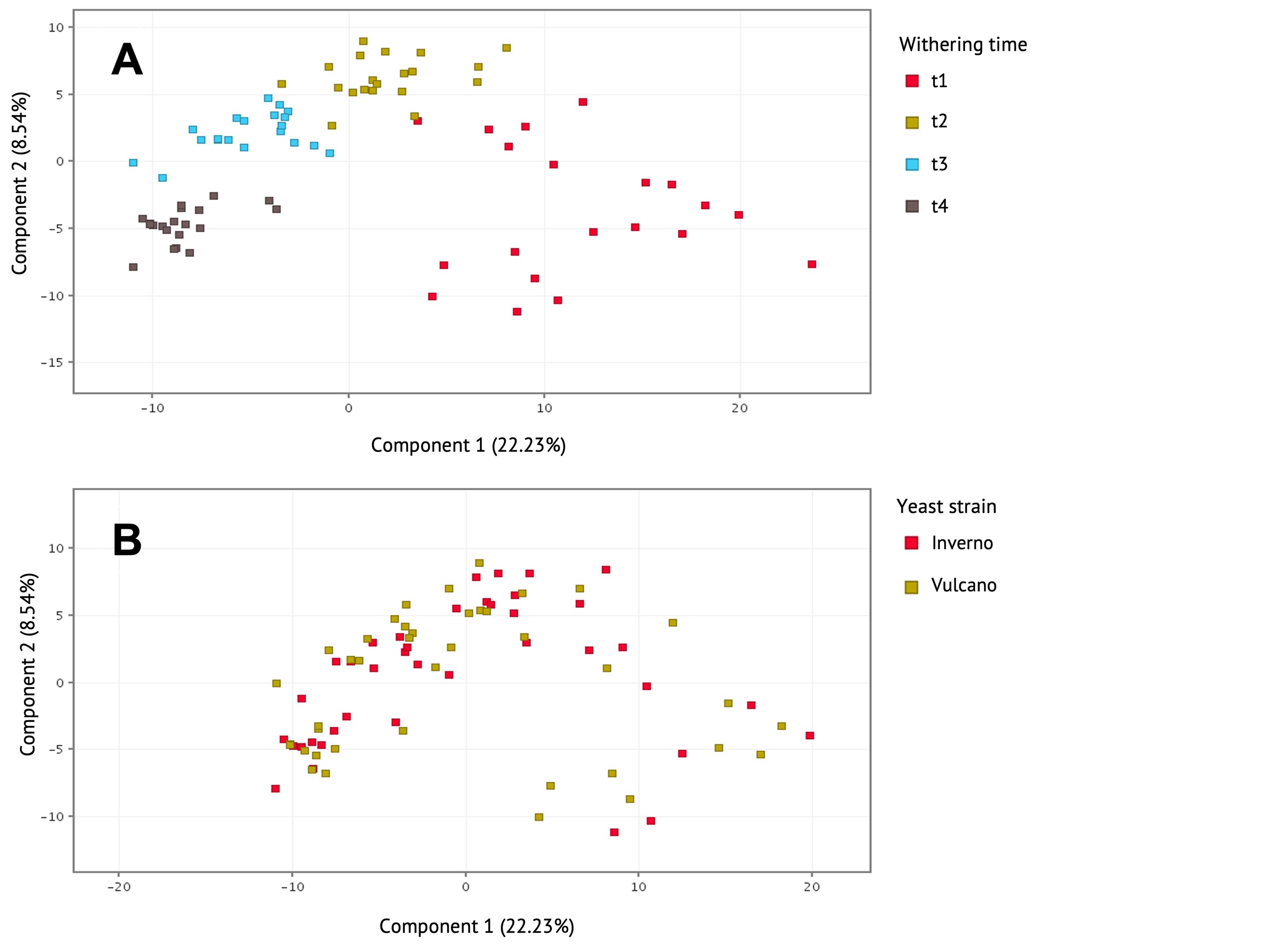


**Figure S2.** Unsupervised principal component analysis (PCA) of LC-HRMS data considering the wine samples (**A**) from different withering time points and (**B**) fermented with the two different yeast strains.

**
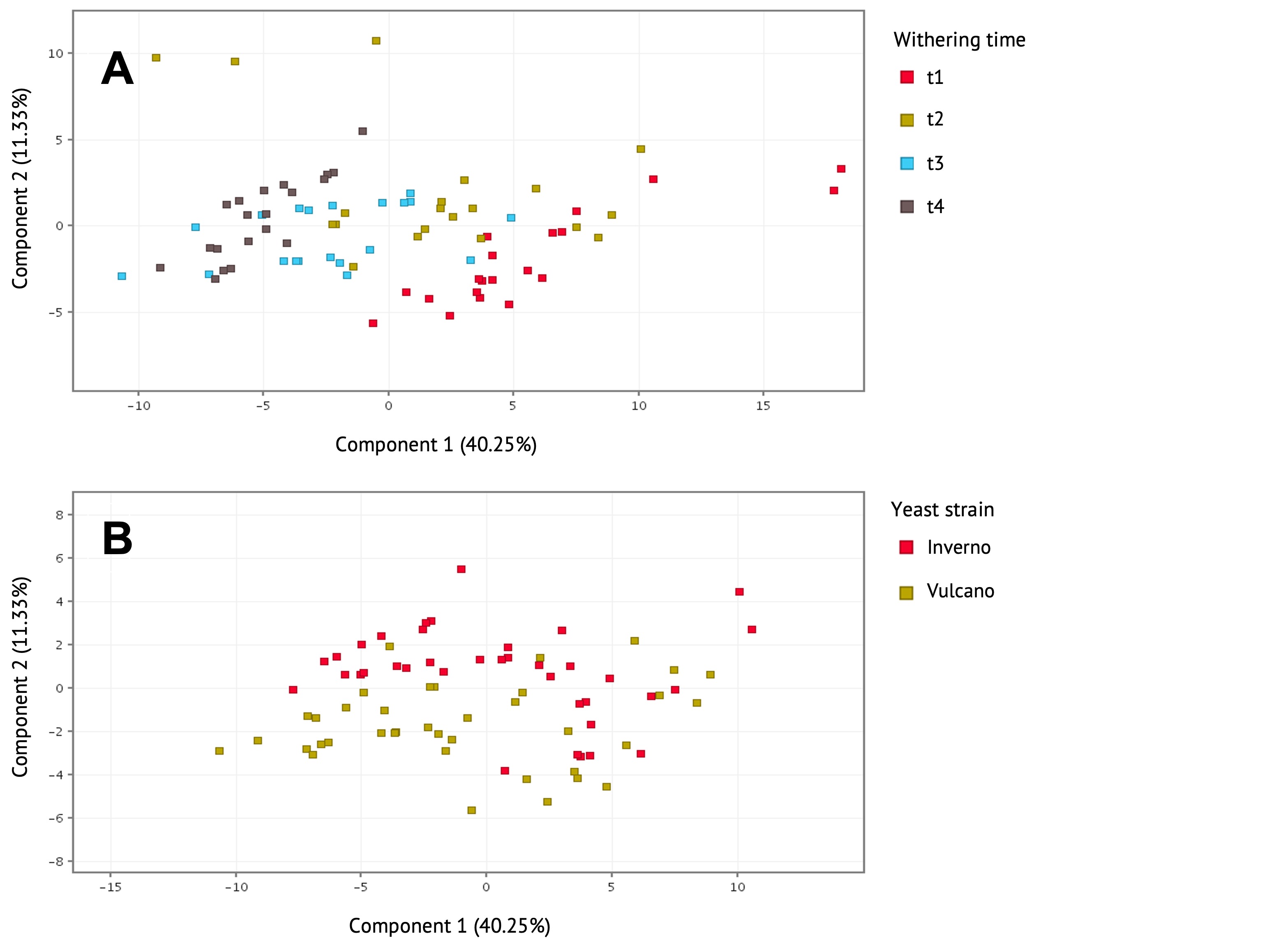
**

**Figure S3.** Unsupervised principal component analysis (PCA) of ^1^H-NMR data considering the wine samples (**A**) from different withering time points and (**B**) fermented with the two different yeast strains.


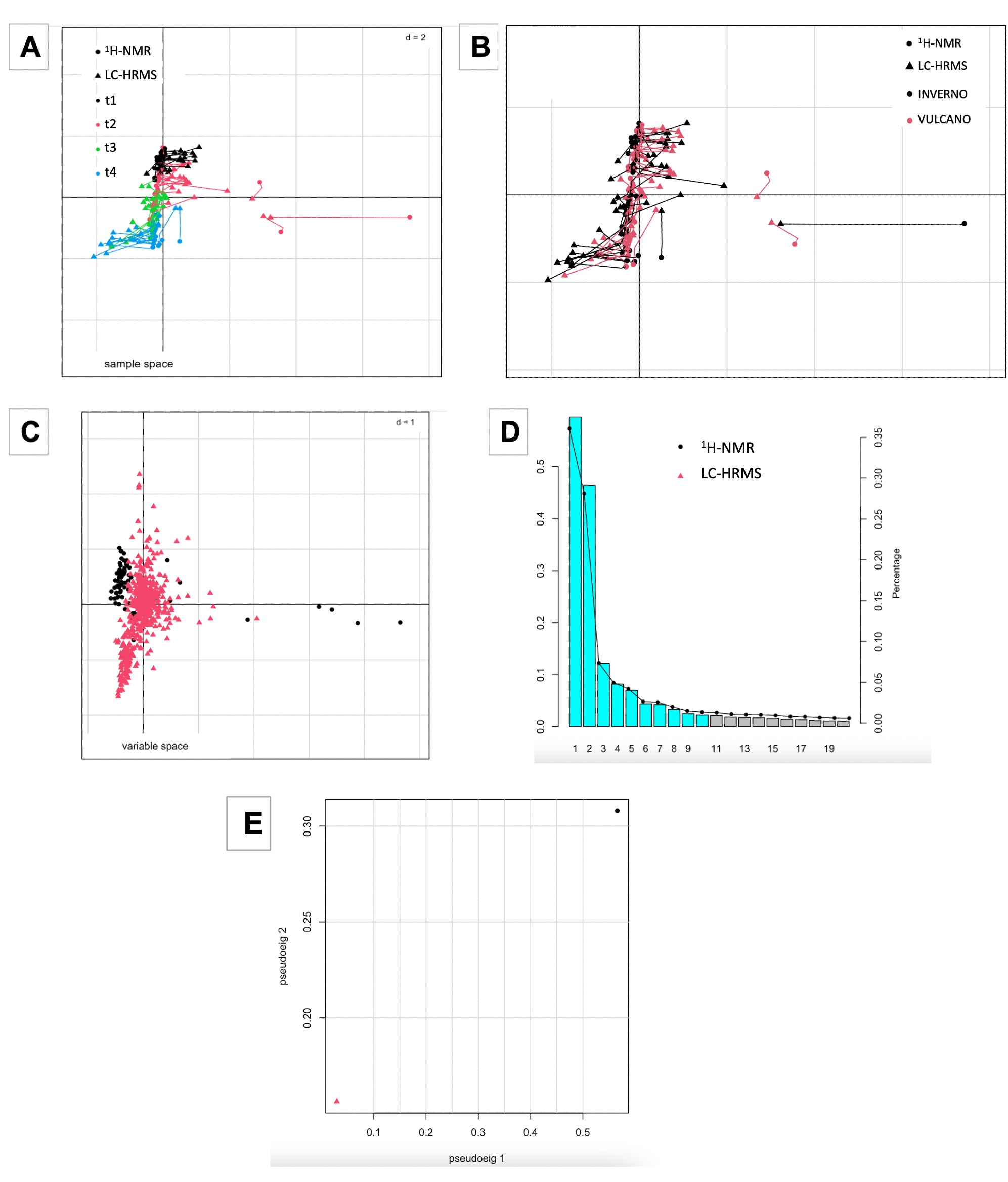


**Figure S4.** MCIA projection plot. The first two components of MCIA that represent LC-HRMS and ^1^H-NMR datasets of Amarone wines according to (**A**) the different withering times of grapes and (**B**) the different yeast strains used during wine fermentation. (**C**) Distribution of the different metabolites defined by the two datasets in the variable space of the first two axes. (**D**) A graphical representation displaying the absolute eigenvalues (represented by bars) and the proportions of variance for the eigenvectors (represented by a line) in a scree plot. (**E**) Summarizing the concordance between datasets by representing pseudo-eigenvalue space.


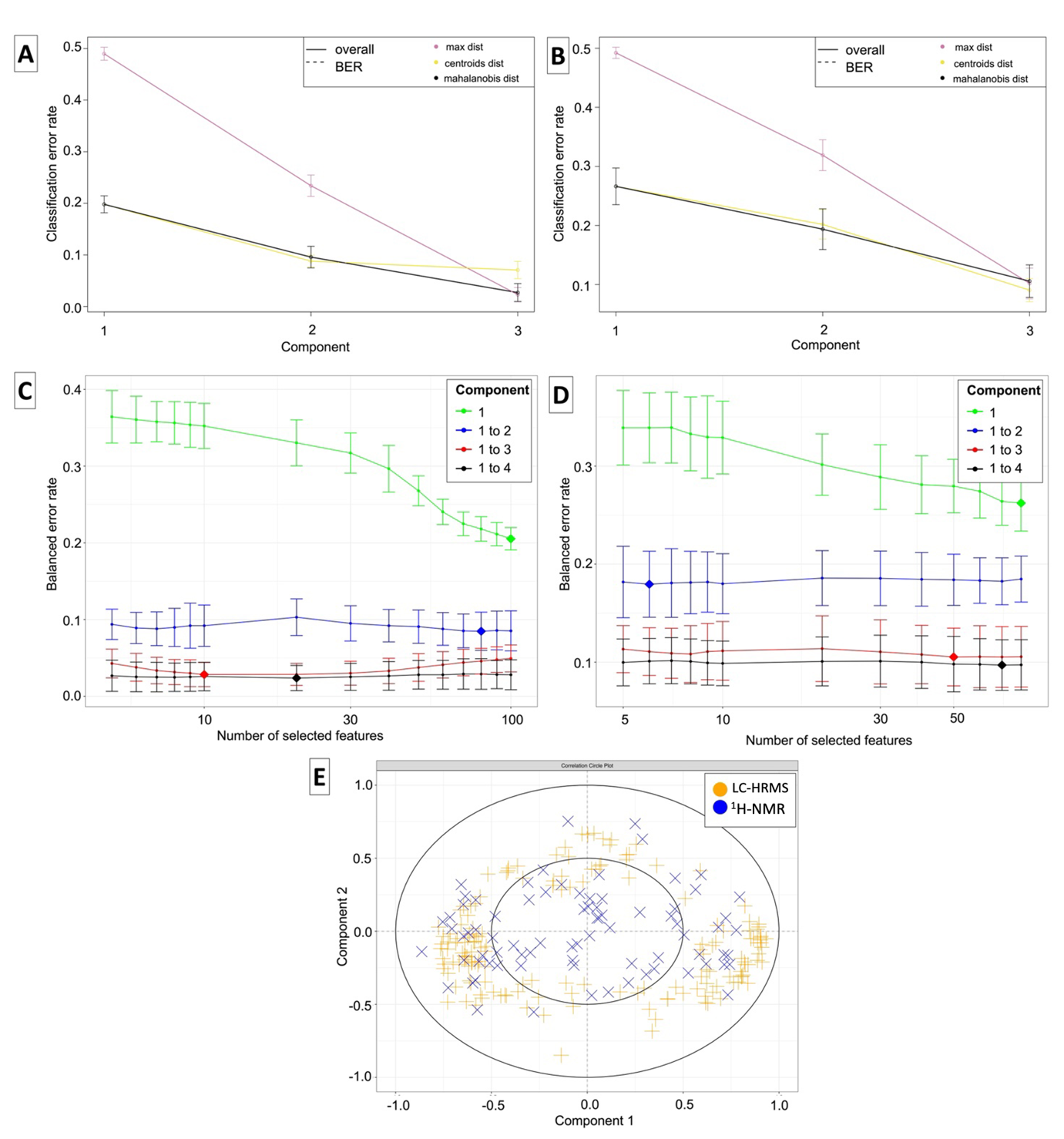


**Figure S5.** Performance of the sparse PLS-DA considering three components in the withering time discrimination for (**A**) LC-HRMS dataset and (**B**) ^1^H-HMR dataset. For each component, repeated cross-validation (50 x 3−fold CV) was used to evaluate the sPLS-DA classification performance (overall and balanced error rate BER), for each type of prediction distance; max.dist, centroids.dist and mahalanobis.dist. Bars show the standard deviation across the repeated folds. Tuning analysis for the sPLS-DA performed on withering time points discrimination for (**C**) LC-HRMS dataset and (**D**) ^1^H-HMR dataset. Each coloured line represents the balanced error rate (y-axis) per component across all tested variable numbers with the standard deviation based on the repeated cross-validation folds. The diamond indicates the optimal number of features on a particular component which achieves the lowest classification error rate as determined with a one-sided t− test. Correlation circle plot of selected features to make integrated sPLS-DA model (**E**).


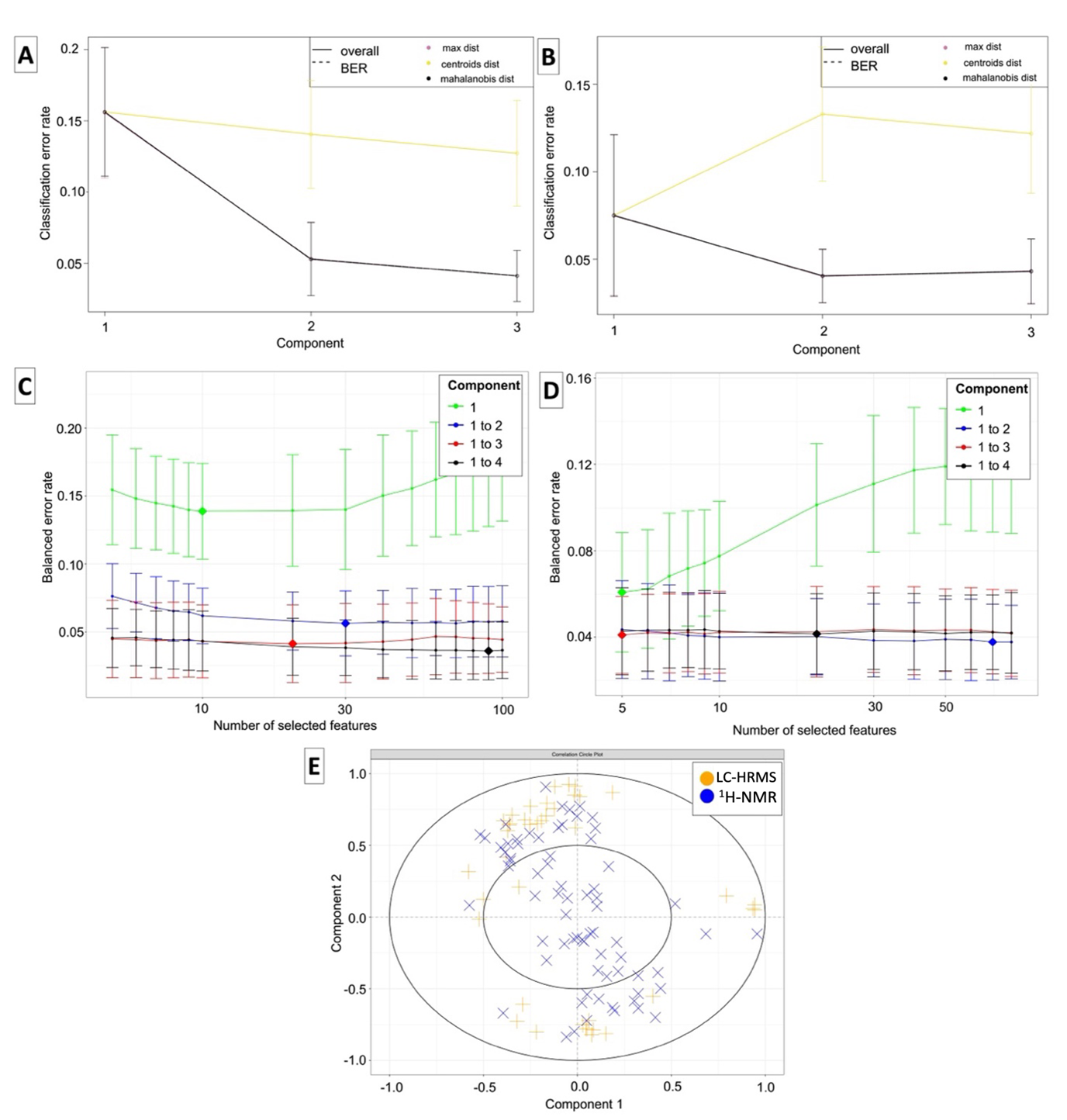


**Figure S6.** Performance of the sparse PLS-DA considering three components in the yeast type discrimination for (**A**) LC-HRMS dataset and (**B**) ^1^H-HMR dataset. For each component, repeated cross-validation (50 x 3−fold CV) was used to evaluate the sPLS-DA classification performance (overall and balanced error rate BER), for each type of prediction distance; max.dist, centroids.dist and mahalanobis.dist. Bars show the standard deviation across the repeated folds. Tuning analysis for the sPLS-DA performed on the yeast type discrimination for (**C**) LC-HRMS dataset and (**D**) ^1^H-HMR dataset. Each coloured line represents the balanced error rate (y-axis) per component across all tested variable numbers with the standard deviation based on the repeated cross-validation folds. The diamond indicates the optimal number of features on a particular component which achieves the lowest classification error rate as determined with a one-sided t-test. Correlation circle plot of selected features to make integrated sPLS-DA model (**E**).


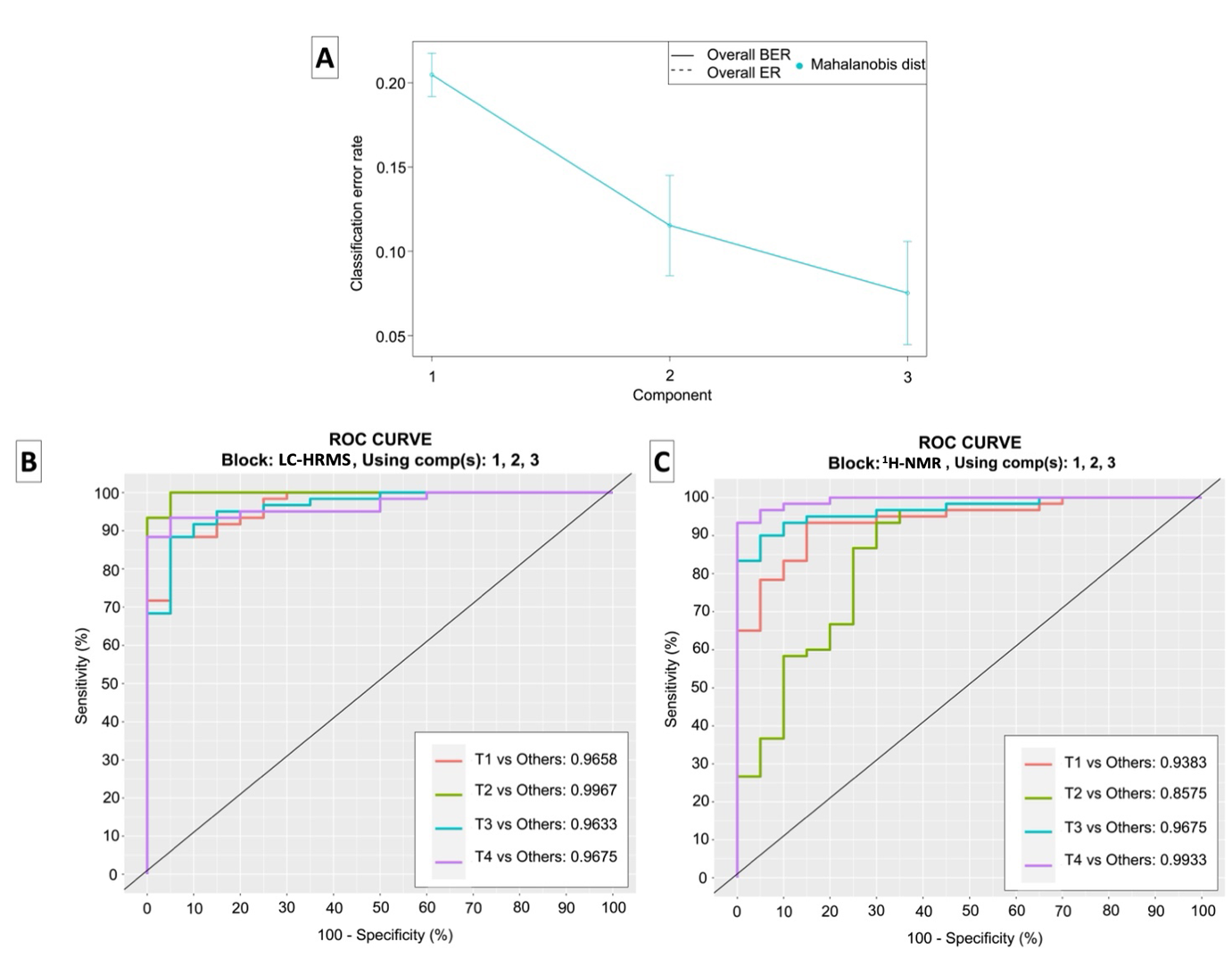


**Figure S7.** Performance of the sparse PLS-DA considering three components in the withering times discrimination for the two considered datasets (**A**). ROC curve and AUC from sPLS-DA on the withering time discrimination data using all the three components averaged across one-*vs*-all comparisons for (**B**) LC-HRMS and (**C**) ^1^H-NMR datasets. Numerical outputs include the AUC value for each one-*vs*-all comparisons that are performed per component.


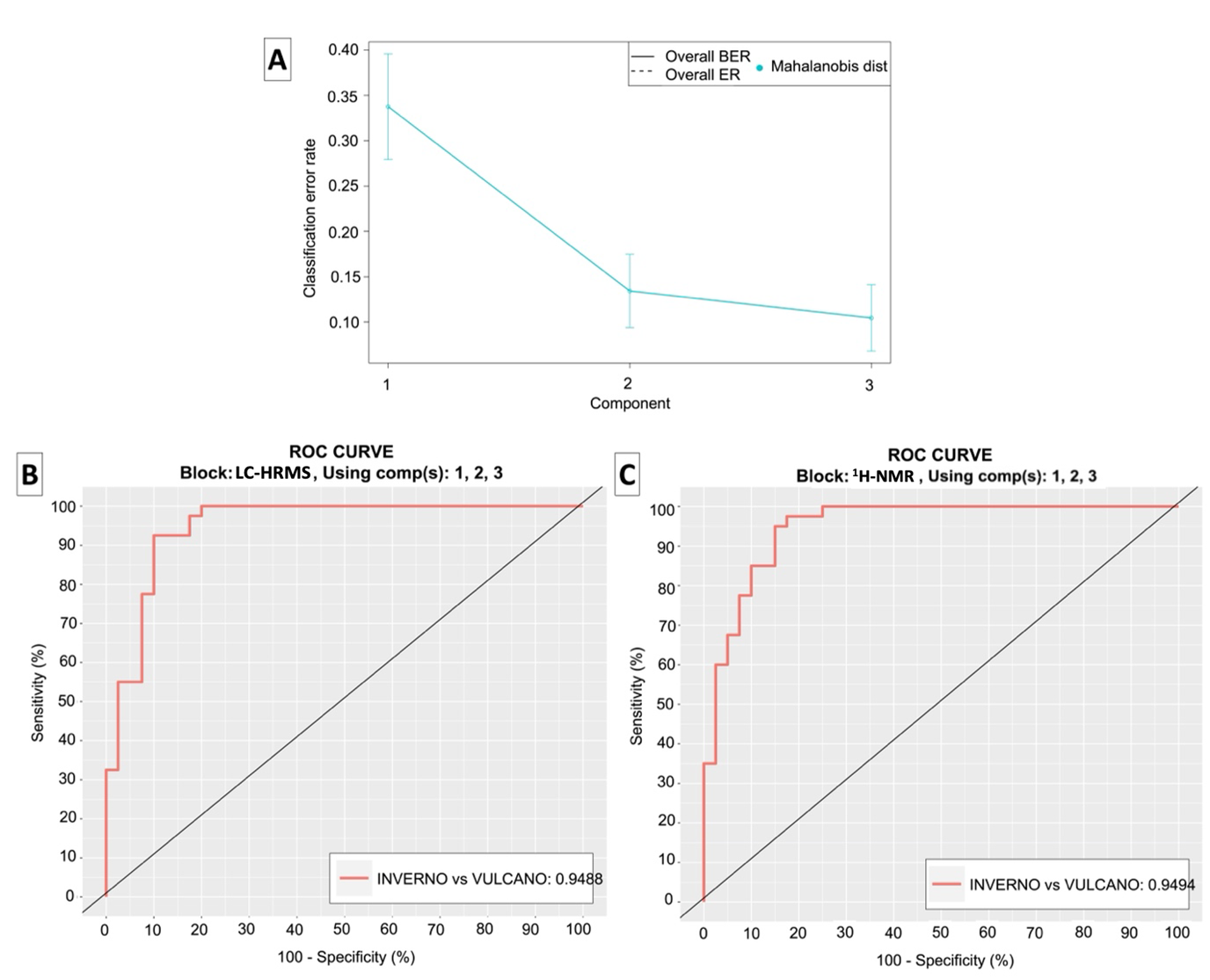


**Figure S8.** Performance of the sparse PLS-DA considering three components in the yeast type discrimination for the two considered datasets (**A**). ROC curve and AUC from sPLS-DA on the yeast type discrimination data using all the three components averaged across one-*vs*-all comparisons for (**B**) LC-HRMS and (**C**) ^1^H-NMR datasets. Numerical outputs include the AUC value for each one-*vs*-all comparisons that are performed per component.
